# Allogeneic hematopoietic cell transplantation after infection with SARS-CoV-2 during the COVID-19 pandemic: a multicenter retrospective analysis

**DOI:** 10.1038/s41409-025-02548-8

**Published:** 2025-03-12

**Authors:** Osama Ahmad, Nicolaus Kröger, Eva Wagner-Drouet, David Nachbaur, Normann Steiner, Daniel Teschner, Sabrina Kraus, Gesine Bug, Salem Ajib, Johannes Schetelig, Wolfgang Andreas Bethge, Thomas Schroeder, Judith Schaffrath, Lutz Peter Müller, Mareike Verbeek, Edgar Jost, Hatice Soysal, Johanna Tischer, Georg-Nikolaus Franke, Stefan Klein, Udo Holtick, Knut Wendelin, Claudia Lengerke, Martin Bornhäuser, Jan Frederic Weller, Maximilian Christopeit, on behalf of the German Cooperative Transplant Study Group, Osama Ahmad, Nicolaus Kröger, Eva Wagner-Drouet, David Nachbaur, Normann Steiner, Daniel Teschner, Sabrina Kraus, Gesine Bug, Salem Ajib, Johannes Schetelig, Wolfgang Andreas Bethge, Thomas Schroeder, Judith Schaffrath, Lutz Peter Müller, Mareike Verbeek, Edgar Jost, Hatice Soysal, Johanna Tischer, Georg-Nikolaus Franke, Stefan Klein, Udo Holtick, Knut Wendelin, Claudia Lengerke, Martin Bornhäuser, Jan Frederic Weller, Maximilian Christopeit

**Affiliations:** 1 Department of Hematology, Oncology, Clinical Immunology and Rheumatology, University Hospital Tuebingen, Tuebingen, Germany; 2 University Medical Center Hamburg, Hamburg, Germany; 3 Department of Hematology, Medical Oncology, and Pneumology, University Medical Center, Mainz, Germany; 4 Department of Internal Medicine V, Hematology and Oncology, Medical University Innsbruck, 6020 Innsbruck, Austria; 5 Division of Hematology, University Hospital of Wurzburg, Wurzburg, Germany; 6 Department of Medicine 2, University Hospital, Goethe University Frankfurt, Frankfurt, Germany; 7 Department of Internal Medicine I, University Hospital Carl Gustav Carus, Technical University Dresden, Dresden, Germany; 8 Department of Hematology and Stem Cell Transplantation, West German Cancer Centre, University Hospital Essen, Essen, Germany; 9 Department of Internal Medicine IV, Hematology and Oncology, Martin Luther University Halle-Wittenberg, Halle, Germany; 10 Department of Medicine III, Hematology and Oncology, Technical University of Munich (TUM), School of Medicine and Health, Munich, Germany; 11 Department of Hematology, Oncology, Hemostaseology and Stem Cell Transplantation, RWTH Aachen University, Medical Faculty, Aachen, Germany; 12 University Hospital Munich-Grosshadern, Department of Internal Medicine III, Ludwig-Maximilian University Munich, Munich, Germany; 13 Department for Hematology, Cell Therapy, Hemostaseology and Infectious Diseases, University of Leipzig Medical Center, Leipzig, Germany; 14 Universitätsmedizin Mannheim, Mannheim, Germany; 15 Faculty of Medicine and University Hospital Cologne, Department I of Internal Medicine, Cologne, Germany; 16 Med Clinic 5, Klinikum Nuremberg, Nuremberg, Germany; 17 University Cancer Center Hamburg, Department of Medicine II - Oncology, Hematology and Bone Marrow Transplantation With Section Pneumology, Hamburg, Germany

**Keywords:** Stem-cell research, Epidemiology, Acute myeloid leukaemia, Infectious diseases

## To the Editor:

At the onset of the COVID-19 pandemic, transplanters faced the unprecedented challenge of balancing the unknown risks of a SARS-CoV-2 infection in patients requiring allogeneic haematopoietic stem cell transplantation (alloHCT) against the benefits of this curative procedure. While alloHCT remains a cornerstone therapy for haematological malignancies, its significant risk of non-relapse mortality (NRM) and prolonged susceptibility to infections make careful timing critical, particularly in the context of modifiable risk factors such as infections [1, 2].

A previous study involving seven patients highlighted the uncertainty early during the pandemic: here transplanters had adopted precautionary measures such as treating patients in insulated areas with dedicated teams [3]. To assess the impact of pre-transplant SARS-CoV-2 infection on alloHCT outcomes, we conducted a retrospective, multicentre study involving 75 patients from 16 centers in Germany and Austria. Patients were categorized into COVID-19 disease severity groups based on WHO criteria. All patients had tested negative for SARS-CoV-2 at the start of conditioning. Severe and critical COVID-19 severity were combined into one group. There were no exclusion criteria.

The study was approved by the local ethics committee of the Eberhard Karls University Tübingen on 26th of May 2021 (project number: 302/2021B02).

Of the 75 patients included, 31 had mild, 6 moderate, and 12 severe/ critical COVID-19. COVID-19 classification of 26 patients remained unknown. The median time from diagnosis of neoplasia to COVID-19 varied between severity groups: 109 days for mild, 374 days for moderate, and 17 days for severe/ critical infections. The median FEV1 prior to alloHCT was 91% in mild and moderate cases, compared to 74% in severe/ critical cases.

The cumulative incidences of relapse and NRM at 365 days for all patients were 17.31% (95% CI: 6.24–28.38) and 8.32% (95% CI: 1.15–15.49), respectively. According to COVID-19 severity, relapse rates were 32.1% for mild, 0% for moderate, and 27.98% for severe/ critical cases, while NRM rates were 0%, 33.33%, and 19.64%, respectively. NRM differences were statistically significant (*p* = 0.03).

Survival analysis revealed significant differences between severity groups. Patients with mild COVID-19 had the highest 365-day survival (90.9%, 95%CI: 79.5–100%), followed by moderate (66.7%, 95% CI: 37.9–100%) and severe/ critical cases (51.1%, 95% CI: 27.9–93.6%). The difference in overall survival (OS) was statistically significant (*p* = 0.025).

When stratifying OS by both COVID-19 severity and Karnofsky Index, patients with mild disease and a high Karnofsky Index (90–100) had the best outcomes (94.4% survival at 365 days). Conversely, patients with severe/ critical COVID-19 and a low Karnofsky Index (<90) had the worst outcomes (51.4% survival at 365 days). Although the combined effect of COVID-19 severity and Karnofsky Index on OS was not statistically significant (*p* = 0.12), these results emphasize the importance of functional assessment in alloHCT candidates.

Univariate Cox regression analysis (Table 1) identified three significant predictors of outcome. First, a high Karnofsky Index (90–100) was associated with significantly better disease-free survival (DFS) compared to a Karnofsky Index <90 (HR: 0.23, 95% CI: 0.07–0.78, *p* = 0.019). Second, patients with severe/ critical COVID-19 had a higher hazard ratio for death compared to those with mild disease (HR: 7.20, 95% CI: 1.39–37.2, *p* = 0.019). Third, a higher FEV1/FVC ratio before alloHCT was associated with a slightly increased hazard for OS and DFS (HR: 1.01, *p* = 0.049 and *p* = 0.028, respectively).

**Table 1:** Univariate cox regression analysis of patient and COVID-19 characteristics on OS and DFS.

Characteristic	Overall survival				Disease-free survival			
	*N*	HR	95% CI	*p*-value	*N*	HR	95% CI	*p*-value
Year of Transplantation	49				49			
2020		—	—			—	—	
2021		0.31	0.08, 1.15	0.080		0.38	0.11, 1.29	0.12
2022		0.00	0.00, Inf	>0.9		0.00	0.00, Inf	>0.9
Disease Status at HSCT	38				38			
CR		—	—			—	—	
no CR		2.97	0.54, 16.2	0.2		0.79	0.14, 4.30	0.8
Karnofsky Index	48				48			
<90		—	—			—	—	
90–100		0.55	0.15, 2.07	0.4		0.23	0.07, 0.78	0.019
Patient Age at HSCT (years)	49	1.01	0.97, 1.06	0.6	49	0.98	0.94, 1.02	0.3
Patient Sex	49				49			
Female		—	—			—	—	
Male		0.92	0.25, 3.45	>0.9		0.63	0.19, 2.09	0.5
Donor Sex	46				46			
Female		—	—			—	—	
Male		0.31	0.08, 1.14	0.079		0.55	0.17, 1.83	0.3
Donor Type	49				49			
Haplo		—	—			—	—	
MMUD		0.88	0.12, 6.25	0.9		1.14	0.23, 5.68	0.9
MRD		0.36	0.03, 3.97	0.4		0.26	0.03, 2.46	0.2
MUD		0.75	0.14, 4.12	0.7		0.58	0.13, 2.64	0.5
CMV Antibody Match	49				49			
Match		—	—			—	—	
IgG negative donor, positive patient		2.61	0.62, 10.9	0.2		1.50	0.40, 5.67	0.5
IgG positive donor, negative patient		0.96	0.11, 8.27	>0.9		0.00	0.00, Inf	>0.9
Karyotyp	41				41			
Abnormal		—	—			—	—	
Normal		0.79	0.14, 4.34	0.8		0.54	0.11, 2.71	0.5
Conditioning	41				41			
MAC		—	—			—	—	
RIC/NMA		2.17	0.44, 10.8	0.3		1.05	0.19, 5.77	>0.9
Maximal Severity of COVID-19 (WHO)	49				49			
Mild		—	—			—	—	
Moderate		4.90	0.69, 34.9	0.11		1.36	0.25, 7.41	0.7
Severe/critical		7.20	1.39, 37.2	0.019		1.99	0.53, 7.52	0.3
Time from Neoplasia to COVID-19 (days)	39	1.00	1.00, 1.00	>0.9	39	1.00	1.00, 1.00	0.10
Time from COVID-19 to HSCT (days)	48	1.00	0.99, 1.01	>0.9	48	1.00	0.99, 1.01	0.7
CT-abnormalities	49				49			
No pneumonia		—	—			—	—	
Pneumonia like changes		3.49	0.44, 27.9	0.2		2.11	0.46, 9.76	0.3
FEV1 (%) prior to Allo	32	0.97	0.91, 1.05	0.5	32	0.98	0.93, 1.04	0.6
FEV1/FVC (%)	39	1.01	1.00, 1.03	0.049	39	1.01	1.00, 1.03	0.028
DLCO (%) prior to Allo	29	0.92	0.85, 1.00	0.056	29	0.98	0.93, 1.03	0.4
ICU during COVID19	46				46			
No		—	—			—	—	
Yes		2.12	0.51, 8.89	0.3		1.74	0.43, 7.02	0.4
GvHD Prophylaxis	36				36			
ATG-based		—	—			—	—	
CsA-based		2.65	0.44, 15.9	0.3		2.12	0.43, 10.5	0.4
postCy-based		2.44	0.34, 17.4	0.4		2.40	0.46, 12.4	0.3
Peak CRP during COVID19 (mg/dl)	26	1.00	0.90, 1.11	>0.9	26	1.02	0.92, 1.12	0.7
Peak IL6 during COVID19 (ng/l)	14	1.00	1.00, 1.00	0.094	14	1.00	1.00, 1.00	0.10
Peak PCT during COVID19 (ng/ml)	13	0.02	0.00, 24.3	0.3	13	0.00	0.00, 67.1	0.3

*HR* Hazard Ratio, *CI* Confidence Interval, *HSCT* Hematopoietic stem cell transplantation, *CR* Complete remission, *MMUD* Mismatched unrelated donor, *MRD* Matched related donor, *MUD* Matched unrelated donor, *CMV* Cytomegalovirus, *IgG* Immunoglobulin G, *MAC* Myeloablative conditioning, *RIC* Reduced intensity conditioning, *NMA* Nonmyeloablative, *WHO* World Health Organization, *CT* Computed tomography, *FEV1* Forced expiratory volume in 1 s, *FVC* Forced vital capacity, *DLCO* Diffusing capacity, *ICU* Intensive care unit, *GvHD* Graft-versus-host disease, *ATG* Antithymocyte globulin, *CsA* Cyclosporine A, *Cy* Cyclophosphamide, *CRP*  C-reactive protein, *IL6* Interleukin 6, *PCT* Procalcitonin.

Our study highlights the pivotal role of COVID-19 severity in determining post-transplant outcomes. Patients with mild COVID-19 demonstrated significantly better OS and lower NRM rates compared to those with severe/ critical disease. These results are consistent with previous reports that severe/ critical COVID-19 exacerbates underlying health conditions, leading to worse prognoses in immunocompromised patients [4–6].

Consistent with prior studies, no significant association between COVID-19 severity and the incidence of Graft-versus-Host Disease (GvHD) was found [3, 7]. The Karnofsky Index emerged as a key independent predictor of survival. Although the combined effect of the Karnofsky Index and COVID-19 severity on survival showed a trend toward association, it did not reach statistical significance. These findings highlight the critical role of functional status assessment in pre-transplant risk stratification.

The evolving impact of improved pandemic management strategies is also evident in our results. By 2022, severe COVID-19 cases were absent from our cohort, likely reflecting the benefits of vaccination, antiviral therapies, and improved infection control measures. These findings highlight the adaptability of transplant practices during this pandemic. They might provide a framework for managing similar crises in the future.

The results of our study should be interpreted with caution due to limitations inherent to the retrospective design. The small sample size and missing data, particularly regarding COVID-19 severity and duration, limit the generalizability of our results. Not all patients were treated for COVID-19 at the participating centers, limiting the availability of consistent data. Future research involving larger cohorts and standardized data collection is needed to validate our findings and explore the mechanisms underlying the observed associations.

In conclusion, our study underscores the profound influence of COVID-19 severity and pre-transplant functional status on alloHCT outcomes. We advocate for continued prioritization of preventive measures, including vaccination and tailored pre-transplant assessments, to optimize patient outcomes in this vulnerable population.

## Data Availability

The datasets generated during and/or analysed during the current study are available from the corresponding author on reasonable request.
